# Insulin modulates cytokine release and selectin expression in the early phase of allergic airway inflammation in diabetic rats

**DOI:** 10.1186/1471-2466-10-39

**Published:** 2010-07-28

**Authors:** Joilson O Martins, Carlos AL Campos, José WMC Cruz, Simone Manzolli, Venâncio AF Alves, Elcio O Vianna, Sonia Jancar, Paulina Sannomiya

**Affiliations:** 1 Institute of Heart (InCor), LIM-11, University of São Paulo Medical School, Av. Dr. Arnaldo, 455 - São Paulo/SP - 01246903 - Brazil; 2 Department of Pathology, University of São Paulo Medical School, Av. Dr. Arnaldo, 455 - São Paulo/SP - 01246903 - Brazil; 3 Department of Immunology, Institute of Biomedical Sciences, University of São Paulo, Av. Prof. Lineu Prestes, 1730 - São Paulo/SP - Brazil; 4 Department of Medicine, Medical School of Ribeirão Preto, University of São Paulo, Av. Bandeirantes, 3900 - Ribeirão Preto/SP - 14049-900 - Brazil

## Abstract

**Background:**

Clinical and experimental data suggest that the inflammatory response is impaired in diabetics and can be modulated by insulin. The present study was undertaken to investigate the role of insulin on the early phase of allergic airway inflammation.

**Methods:**

Diabetic male Wistar rats (alloxan, 42 mg/Kg, i.v., 10 days) and controls were sensitized by s.c. injection of ovalbumin (OA) in aluminium hydroxide 14 days before OA (1 mg/0.4 mL) or saline intratracheal challenge. The following analyses were performed 6 hours thereafter: a) quantification of interleukin (IL)-1β, tumor necrosis factor (TNF)-α and cytokine-induced neutrophil chemoattractant (CINC)-1 in the bronchoalveolar lavage fluid (BALF) by Enzyme-Linked Immunosorbent Assay, b) expression of E- and P- selectins on lung vessels by immunohistochemistry, and c) inflammatory cell infiltration into the airways and lung parenchyma. NPH insulin (4 IU, s.c.) was given i.v. 2 hours before antigen challenge.

**Results:**

Diabetic rats exhibited significant reduction in the BALF concentrations of IL-1β (30%) and TNF-α (45%), and in the lung expression of P-selectin (30%) compared to non-diabetic animals. This was accompanied by reduced number of neutrophils into the airways and around bronchi and blood vessels. There were no differences in the CINC-1 levels in BALF, and E-selectin expression. Treatment of diabetic rats with NPH insulin, 2 hours before antigen challenge, restored the reduced levels of IL-1β, TNF-α and P-selectin, and neutrophil migration.

**Conclusion:**

Data presented suggest that insulin modulates the production/release of TNF-α and IL-1β, the expression of P- and E-selectin, and the associated neutrophil migration into the lungs during the early phase of the allergic inflammatory reaction.

## Background

Hormones and other endocrine factors play a modulating role in allergic inflammation, with the effects of glucocorticoids or adrenergic agents being obvious examples. It has been already demonstrated that alloxan-induced diabetic rats present markedly reduced inflammatory reactions to allergen challenge in the airways [1,2] and in the pleural space [3] unrelated to changes in the number of total blood leukocytes or blood glucose levels [1], but ascribed to a reduction in mast cell degranulation upon antigen challenge [3-5]. Treatment of diabetic rats with insulin restores the number of degranulated mast cells, levels of histamine release, and airway reactivity to ovalbumin [5]. Because of that, we decided to further investigate the early phase of allergic airway inflammation, which steps of the cell migration process are impaired in diabetic rats, and at what level is insulin acting to restore it.

It has long been recognized that the inflammatory response in diabetic patients is impaired [6-12]. Abnormalities of neutrophil function have been shown to occur during inflammation in alloxan-induced diabetic rats [6,7]. These include: decreased leukocyte-endothelial cell interactions [10,13] and reduced number of leukocytes in inflammatory lesions [10,14-16]; reduced superoxide generation and tumor necrosis factor (TNF)-α release [17]; reduced lymph node retention capacity [18]; a decrease in the generation of prostaglandin (PG)-E_2 _[19]; reduced production/release and transcription of pro-inflammatory (interleukin (IL)-1β, TNF-α), and anti-inflammatory (IL-10) cytokines, and reduced expression of intercellular adhesion molecule (ICAM)-1 [7,8]. Alloxan, currently used to induce diabetes in experimental animals, acts through selective uptake by low affinity GLUT2 glucose transporter into the beta-cell leading to the destruction of the transporter protein by oxygen free radicals [20,21]. It was demonstrated that, in addition to the significant reduction in body weight gain and hyperglycemia, polydipsia, polyuria, glycosuria, presence of ketone bodies, hypoinsulinemia, and elevated levels of glycosylated haemoglobin are present in Wistar rats after alloxan injection [7-16]. The aim of the present study was to compare alloxan-induced diabetic rats with non-diabetics for the levels of TNF-α, IL-1β, and cytokine-induced neutrophil chemoattractant (CINC)-1, in the bronchoalveolar lavage fuid (BALF), and the expression of E and P selectins in lung tissue during the early phase of the allergic lung inflammation. Furthermore, we evaluated the effect of insulin treatment of diabetic rats on these parameters. Data presented show that insulin restored the deficient neutrophil migration observed in diabetic rats in response to allergen challenge. This occurred in parallel with increased TNF-α and IL-1β levels in BALF, and increased expression of E- and P- selectins.

## Methods

### Animals

Male Wistar rats weighing 200 ± 20 g (about 2 months of age) at the beginning of the experiments were used. The animals were maintained at 23 ± 2°C under a cycle of 12 hours light: 12 hours darkness and were allowed access to food and water *ad libitum*. All experiments were in accord with ethical principles in animal research adopted by the Brazilian College of Animal Experimentation. Approval of the Animal Subject Committee of the Heart Institute (InCor), University of São Paulo Medical School, was obtained prior to initiating the experiments.

### Induction of diabetes mellitus and insulin treatment

Diabetes mellitus was induced by an intravenous injection of 42 mg/kg of alloxan monohydrate (Sigma Chemical Co., St. Louis, MO, USA) dissolved in physiologic saline (0.9% NaCl) [7-9,17-21]. Control rats were injected with physiologic saline only. Ten days thereafter blood glucose concentrations were determined and animals that presented glucose above 200 mg/dL were used. Blood samples were obtained from the cut tip of the rat tail and glucose determined with the aid of a blood glucose monitor (Eli Lilly, São Paulo, SP, Brazil). A group of diabetic rats received 4 IU s.c. of neutral protamine Hagedorn (NPH) insulin (Eli Lilly, São Paulo, SP, Brazil) 2 hours before ovalbumin (OA, grade III; Sigma Chemical Co.) challenge. Because the maximum serum concentration (Cmax) of NPH insulin is reached between 6 and 8 h after subcutaneous administration, we decided to give insulin to the animal 2 h before OA challenge, as in previous studies in this line of insulin treatment [7-9]. The dose of insulin injection was chosen based on previous studies from our group [7-9] because the dose was able to restore inflammatory parameters that are suppressed in diabetic rats. Although this dose was not sufficient to return glucose levels in animals with diabetes to normal values, the serum insulin was above normal levels during the time of the experiment (6 h after OA challenge) [7-9,17-19].

### Sensitization to ovalbumin and antigen challenge

The animals were actively immunized with ovalbumin (OA) by subcutaneous injection of 0.2 mL sterile physiologic saline containing 0.1 mg OA and 8 mg Al(OH)_3 _[1]. The animals were anesthetized 14 days thereafter with an intraperitoneal injection (150 mg/Kg) of S (+)-ketamine hydrochloride (Ketamin-S(+), Cristalia, Itapira, SP, Brazil) and the trachea was exposed through a midline ventral incision of approximately 0.5 cm length in the neck. Physiologic saline solution (0.9% NaCl; 0.4 mL) containing 1 mg OA was instilled into the airways. The incision was closed with sutures and the animals returned to their cages.

### Bronchoalveolar lavage fluid

BALF was performed 6 hours after the intratracheal administration of OA or saline. The animals were anesthetized, as described above, and the abdominal cavity was opened for blood exsanguination from the abdominal aorta. The lungs were then lavaged by instillation of 10 mL of phosphate buffered saline (PBS), at room temperature, through a polyethylene tube (1 mm in diameter) inserted into the trachea. The first 10 mL instilled into the lungs were withdrawn and reinstilled twice. After centrifugation (500 g for 15 min), the supernatant was frozen at -70°C for cytokine measurements. The lungs were further lavaged with 15 mL PBS (3 times 5 mL) to harvest leukocytes. The BALF was not used if the retrieved volume was less than 85% of the 25 mL instilled. Total cell counts were determined by using an automatic hemacytometer (CELM, São Paulo, SP, Brazil). Differential cell counts were carried out on hematoxylin-eosin stained slides under oil immersion microscopy. A total of 100 cells were counted and classified as neutrophils, eosinophils or mononuclear cells based on morphologic criteria.

### Lung morphometric analysis

For the analysis of cell infiltration around blood vessels and bronchi animals were anesthetized, the chest wall was opened and the lungs removed. A portion of the left inferior lobe close to the hilum and a portion of the apex were removed, fixed in 4% paraformaldehyde solution in sodium phosphate buffer, 0.2 M, pH 7.2, for 24 h, and then dehydrated in ethanol. Samples were embedded in paraplast embedding media (Sigma Chemical Co., St. Louis, MO), seccioned at 5 μm, and stained with hematoxylin and eosin (Merk, Darmstadt, Germany). Morphometric analysis of perivascular and peribronchiolar infiltration of mononuclear cells, neutrophils and eosinophils were performed in the lung sections. Determinations were made in 3 randomly selected fields close to the vessel (4 vessels/sample) and in the surrounding airway tissue (4 bronchi/sample), by employing a 10^4 ^μm^2 ^total area reticle. Data were then averaged for each animal.

### Quantification of cytokines in the BALF

The concentrations of IL-1β, TNF-α and CINC-1 in the BALF supernatant were determined by enzyme-linked immunosorbent assay (ELISA) using commercially available kits according to the manufacturer's instructions (R & D Systems Inc., Minneapolis, MN, USA). The sensitivity of the assays was 15 pg/mL.

### Immunohystochemistry for E- and P- selectin

Six hours after OA or saline intratracheal challenge, the animals were anesthetized and exsanguinated as described above. After tracheostomy, 10 mL of tissue freezing medium (Leica Instruments Gmbh, Nussloch, Germany) diluted in PBS (1:1, v/v) were instilled into the airways. The lungs were harvested through a median sternotomy, immersed in hexan and frozen into liquid nitrogen. Serial 8 μm cryostat sections were adhered to glass slides previously coated with organo-sylane (Sigma Chemical Co., St. Louis, MO, USA). For the immunodetection of both E- and P- selectins on lung microvessels, a biotin conjugated anti-rat E-selectin (CD62E) polyclonal antibody (R & D Systems Inc., Minneapolis, MN, USA) or anti-human P-selectin (CD62P) monoclonal antibody (Immunotech, Marseille, France) were used. After fixation in acetone, tissue samples were incubated with the antibody overnight at 4°C, rinsed in PBS and treated with streptoavidin-fluorescein (Amersham Pharmacia Biotech, London, UK) for 1 hour at room temperature. After washing the slides with PBS, samples were treated with Vectashield mounting medium containing propidium iodide (Vector, CA, USA) in order to preserve the fluorescence. Negative control samples were incubated with PBS instead of the primary antibody. The system used for image acquisition include the Coosnap Pro Color digital camera (Nikon, Tokyo, Japan), coupled to a triocular fluorescent microscope (Nikon), and Software Image-Pro^® ^Plus, version 4.1 (Media Cybernetics, Silver Spring, MD, USA). Results are presented as mean fluorescence intensity.

### Statistical analysis

Data are presented as means ± SEM and analyzed by ANOVA followed by the Tukey-Kramer multiple comparisons test. *p *> 0.05 was considered significant.

## Results

### Body weight gain and blood glucose levels

Table 1 shows that, relative to controls, animals rendered diabetic by injection of alloxan exhibited a significant reduction in body weight gain during the experimental period, and sharply elevated blood glucose levels. Treatment of diabetic animals with a single dose of NPH insulin induced a significant reduction in blood glucose levels but it was not sufficient to reduce glycemia to control values.

**Table 1:** General characteristics of the animals

Groups	Body weight gain (g)	Blood glucose (mg/dL)
Control	147 ± 12	87 ± 2
Diabetic	11 ± 11*	531 ± 26*
Diabetic + insulin	13 ± 12 *	257 ± 15 *^†^

Rats were rendered diabetic by the injection of alloxan (42 mg/Kg, i.v.), and measurement of body weight was carried out 24 days afterwards. Rats were immunized with OA and blood glucose levels in the 3 groups were determined 6 hours after antigen challenge. Insulin (NPH, 4 IU/rat, s.c.) was administered 2 hours before the intratracheal instillation procedure.Values are means ± SEM. The number of animals in each group was 9. *P < 0.001 comparing diabetic with control (non-diabetic) rats. ^†^*P *< 0.001 comparing insulin-treated *vs *non-treated diabetic rats.

### Role of insulin on leukocyte migration

Relative to control (non-diabetic) OA-challenged rats, leukocyte counts in the BALF of diabetic rats were reduced after OA challenge due to a 90% reduction in the number of neutrophils, and 50% reduction in the number of mononuclear cells. Treatment of diabetic animals with a single dose of NPH insulin, 2 hours before OA challenge, restored the impaired cell migration observed in diabetic rats to values attained in control non-diabetis rats. Only few eosinophils were found and there were no differences among groups (Table 2). The morphometric analysis of lung parenchyma showed that in both, diabetic and non diabetic rats, the allergic reaction induced cell infiltration around blood vessels and bronchi 6 h after challenge. However, diabetic rats exhibited a 40% reduction in the number of neutrophils, and a 25% reduction in the number of mononuclear cells in both perivascular and peribronchiolar sites. Treatment of diabetic rats with 4 IU NPH insulin before OA challenge restored the cell migration to the levels found in control non-diabetic rats. The number of eosinophils was very low and did not differ among groups (Table 2).

**Table 2:** Effect of insulin on the inflammatory cells infiltration into the airways and lung parenchyma

	Control (non-diabetic)	Diabetic	Diabetic +insulin
**BALF (Cells × 10^6^)**			
Mononuclears	14.28 ± 0.58	7.11 ± 0.18 *	13.08 ± 0.74 **^†^**
Neutrophils	9.63 ± 0.43	0.94 ± 0.11 *	9.51 ± 0.58 **^†^**
Eosinophils	0.04 ± 0.02	0.02 ± 0.01	0.03 ± 0.01
**Perivascular (Cells/10^4 ^μm^2^)**			
Mononuclears	74.54 ± 2.65	56.75 ± 1.72 *	66.18 ± 2.15 **^†^**
Neutrophils	26.97 ± 1.56	15.58 ± 1.46 *	33.13 ± 3.75 **^†^**
Eosinophils	0.40 ± 0.16	0.50 ± 0.15	0.35 ± 0.13
**Peribronchiolar (Cells/10^4 ^μm^2^)**			
Mononuclears	75.38 ± 2.90	56.30 ± 1.79 *	67.50 ± 3.10 **^†^**
Neutrophils	27.63 ± 2.44	18.08 ± 1.79 *	32.12 ± 3.23 **^†^**
Eosinophils	0.46 ± 0.24	0.25 ± 0.11	0.35 ± 0.17

Rats were rendered diabetic by the injection of alloxan (42 mg/Kg, i.v.) 10 days before sensitization. In both non-diabetic and diabetic rats, active sensitization against ovalbumin (OA) was performed 14 days before OA intratracheal instillation. Insulin (NPH, 4 IU/rat, s.c.) was administered 2 hours before OA challenge. Bronchoalveolar lavage fluid (BALF) was performed 6 hours thereafter (9 animals in each group). Morphometric analysis was made in the stained slides of lung tissue. Determinations were made in 3 randomly selected fields for the presence of cells in the perivascular (4 vessels/sample) and peribronchiolar (4 bronchi/sample) tissue (3 animals in each group). Values are means ± SEM. *P < 0.001 comparing diabetic with control (non-diabetic) rats. ^†^*P *< 0.001 comparing insulin-treated *vs *non-treated diabetic rats.

### Role of insulin on cytokines levels

**Figure 1:**
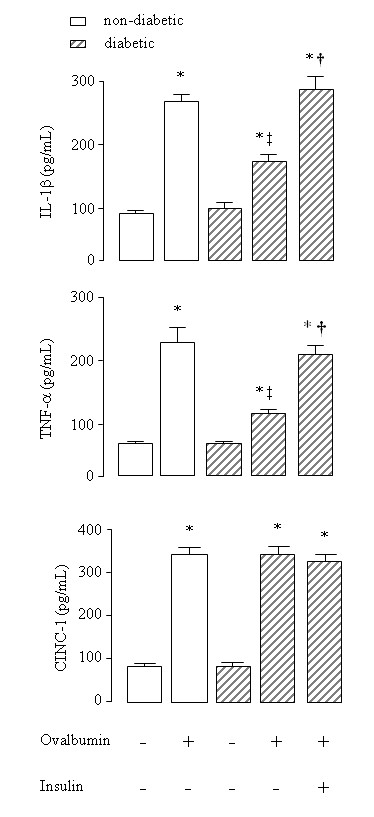
**IL-1β, TNF-α and CINC-1 concentrations in the bronchoalveolar lavage fluid of ovalbumin (OA) sensitized non-diabetic and diabetic rats 6 hours after OA (experimental) or saline (control) intratracheal instillation**. Insulin (NPH, 4 IU/rat s.c.) was given to diabetic rats 2 hours before OA challenge. Values are means ± SEM for 5 to 7 animals in each group. *P < 0.001 comparing OA challenged with the control in the corresponding group (diabetic or non-diabetic). ^†^*P *< 0.0001 comparing diabetic rats treated *vs *non-treated with insulin. ^‡^*P *< 0.001 comparing OA challenged with the diabetic or non-diabetic group.

In the BALF of non-diabetic rats a 3.5-fold increase in the concentration of IL-1β and a 4.3-fold increase in the concentration of TNF-α were observed after instillation of OA into immunized rats compared to saline instilled rats. In contrast, a reduction in the levels of IL-1β (38%) and TNF-α (54%) were observed in OA-instilled diabetic rats compared to OA-instilled non-diabetic rats. Results are illustrated in Figure 1. Levels of these cytokines were similar in saline-instilled diabetic and non-diabetic rats. Treatment of diabetic rats with a single dose of NPH insulin, 2 hours before OA challenge completely restored BALF IL-1β and TNF-α levels (Figure 1). A 3-fold increase in the BALF concentrations of CINC-1 was observed after exposure of non-diabetic rats to OA compared to saline-instilled rats. Similar results were observed in diabetic rats after OA challenge compared to saline-instilled diabetic rats (Figure 1). Levels of CINC-1 were similar in saline-instilled diabetic and non-diabetic animals. Furthermore, CINC-1 levels did not change in diabetic rats after treatment with a single dose of NPH insulin, 2 hours before OA challenge (Figure 1).

### Role of insulin on lung adhesion molecules

**Figure 2:**
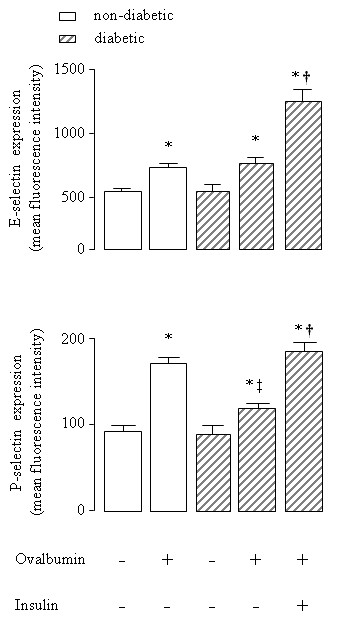
**Quantitative evaluation of the immune staining for E- and P- selectins on lung microvessels of ovalbumin (OA) sensitized non-diabetic and diabetic rats 6 hours after OA (experimental) or saline (control) instillation**. Insulin (NPH, 4 IU/rat s.c.) was given to diabetic rats 2 hours before OA challenge. Values are means ± SEM for 8 samples/rat, 3 animals/group. Analyses were performed by using the software image-pro Plus, version 4.1, Media Cybernetics. *P < 0.001 comparing OA challenged with the control in the corresponding group (diabetic or non-diabetic). ^†^*P *< 0.0001 comparing diabetic rats treated *vs *non-treated with insulin. ^‡^*P *< 0.001 comparing OA challenged with the diabetic or non-diabetic group.

**Figure 3:**
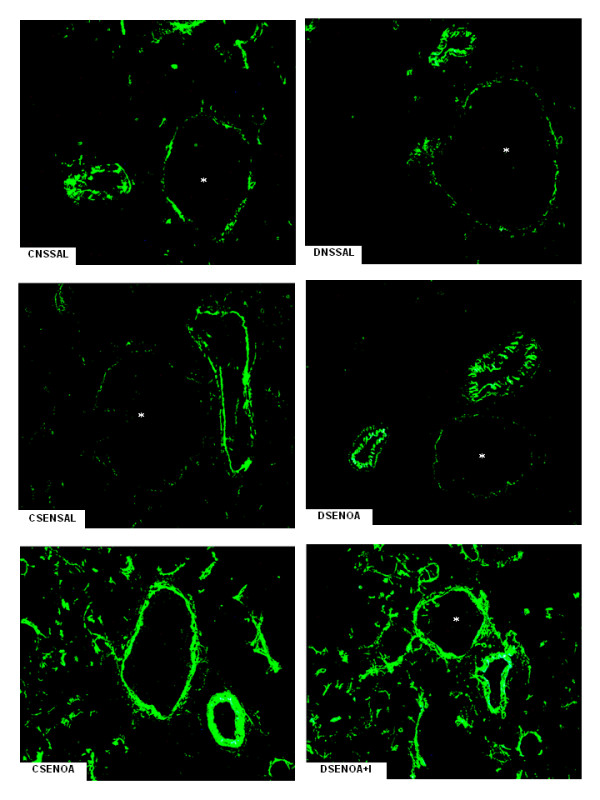
**E-selectin lung tissue microphotographs**. The immune staining for E-selectin on lung microvessels of ovalbumin (OA) sensitized non-diabetic and diabetic rats 6 hours after OA (experimental) or saline (control) instillation. Insulin (NPH, 4 IU/rat s.c.) was given to diabetic rats 2 hours before OA challenge. Values are means ± SEM for 8 samples/rat, 3 animals/group. Analyses were performed by using the software image-pro Plus, version 4.1, Media Cybernetics. The microphotographs of lung tissue were obtained from control non-diabetic rats non-sensitized and instilled with saline (CNSSAL) or sensitized and instilled with saline (CSENSAL) or sensitized and instilled with OA (CSENOA), diabetic rats non-sensitized instilled with saline (DNSSAL) or sensitized and instilled with OA (DSENOA), and insulin treated diabetic rats sensitized instilled with OA (DSENOA+I). *Indicates the vessel lumen. Lung sections (8 μm) were stained for the detection of E-selectin (original magnification × 1500). *P < 0.001 comparing OA challenged with the control in the corresponding group (diabetic or non-diabetic). ^†^*P *< 0.0001 comparing diabetic rats treated *vs *non-treated with insulin. ^‡^*P *< 0.001 comparing OA challenged with the diabetic or non-diabetic group.

**Figure 4:**
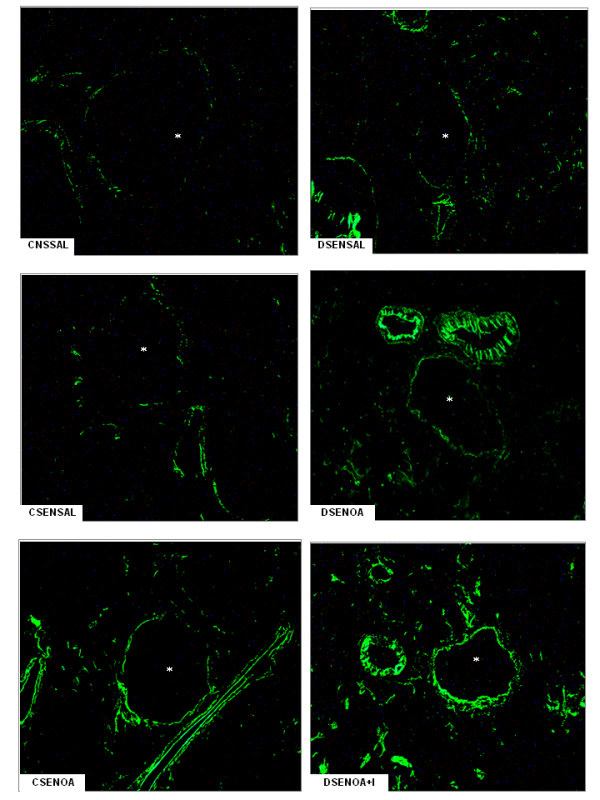
**P-selectin lung tissue microphotographs**. The immune staining for P-selectin on lung microvessels of ovalbumin (OA) sensitized non-diabetic and diabetic rats 6 hours after OA (experimental) or saline (control) instillation. Insulin (NPH, 4 IU/rat s.c.) was given to diabetic rats 2 hours before OA challenge. Values are means ± SEM for 8 samples/rat, 3 animals/group. Analyses were performed by using the software image-pro Plus, version 4.1, Media Cybernetics. The microphotographs of lung tissue were obtained from control non-diabetic rats non-sensitized and instilled with saline (CNSSAL) or sensitized and instilled with saline (CSENSAL) or sensitized and instilled with OA (CSENOA), diabetic rats non-sensitized instilled with saline (DNSSAL) or sensitized and instilled with OA (DSENOA), and insulin treated diabetic rats sensitized instilled with OA (DSENOA+I). *Indicates the vessel lumen. Lung sections (8 μm) were stained for the detection of P-selectin (original magnification × 1500). *P < 0.001 comparing OA challenged with the control in the corresponding group (diabetic or non-diabetic). ^†^*P *< 0.0001 comparing diabetic rats treated *vs *non-treated with insulin. ^‡^*P *< 0.001 comparing OA challenged with the diabetic or non-diabetic group.

E-selectin expression, as evidenced by immune staining, increased 1.5-fold in lung microvessels of sensitized non-diabetic rats 6 hours after OA challenge compared to saline-instilled rats. Similar results were observed in OA-challenged diabetic rats compared to saline-instilled diabetic rats. Despite no significant differences between non-diabetic and diabetic animals, expression of E-selectin was increased (77%) after insulin treatment of diabetic rats. Quantitative evaluation of the immune staining is illustrated in Figure 2. Representative sections of these preparations are shown in Figure 3. Levels of E-selectin were not significantly different between saline-instilled diabetic and non-diabetic rats. Expression of P-selectin on lung microvessels of non-diabetics increased 1.8-fold after exposure to OA compared to saline-instilled rats. In contrast, in diabetic rats the increase in P-selectin in response to allergic stimulation was only 1.3-fold. Treatment of diabetic rats with a single dose of NPH insulin, 2 hours before OA challenge completely restored P-selectin expression Quantitative evaluation of the immune staining is illustrated in Figure 2. Representative sections of these preparations are shown in Figure 4.

## Discussion

Results presented here suggest that insulin modulates the development of the inflammatory reaction to allergen challenge by its ability to regulate the production of IL-1β and TNF-α and the expression of P-selectin. This is supported by the following observations: (a) relative to control non-diabetic rats, alloxan-induced diabetic rats exhibited reduced levels of IL-1β and TNF-α in the BALF and reduced lung P-selectin expression; (b) this was accompanied by a significant reduction in the number of neutrophils and mononuclear leukocytes harvested from the BALF and infiltrating perivascular and peribronchiolar tissues; (c) a complete recovery of the impaired responses was observed under the influence of insulin; (d) despite no significant difference in lung E-selectin expression between diabetic and non-diabetic rats, an increase in the expression of this adhesion molecule was observed after insulin treatment.

In a previously published study, Vianna and Garcia-Leme have demonstrated that insulin modulates leukocyte migration into the airways during the course of the allergic reaction [1]. In agreement with their findings, we found that the number of neutrophils and mononuclear cells in the BALF of diabetic rats was markedly reduced in response to OA challenge. In addition, reduced numbers of neutrophils and mononuclear leukocytes were observed surrounding blood vessels and bronchi of diabetic rats. The impaired response was apparently not related to antibody production or to blood glucose levels [1] but ascribed to a reduction in mast cell degranulation upon antigen challenge [5]. Indeed, it has been reported that mast cells from diabetic rats were shown to be refractory to antigen stimulation both in vivo [3] and in vitro [4].

Reversal of the impaired responses was attained by treatment of diabetic rats with insulin. Despite an average of 50% reduction in the levels of blood glucose after treatment with a single dose of insulin, diabetic rats are still hyperglycemic compared to controls. Insulin in adequate concentrations seems to be required for the normal function of alveolar macrophages [22,23], endothelial cells and neutrophils during the course of the inflammatory process [6]. Moreover, levels of serum corticosterone did not differ significantly between diabetic rats and controls [7]. Accordingly, the suggestion is that the restorative effect observed in insulin-treated rats might be primarily linked to a continuing insulin deficiency rather than to the hyperglycaemic state of diabetic animals, or to a prevalence of glucocorticoids.

On activation, mast cells rapidly synthesize bioactive metabolites of arachidonic acid, prostaglandins and leukotrienes, and many mediators are released by triggered exocytosis from intracellular stores, including histamine, numerous proteases, and TNF-α [3-5,24]. Gene expression is also activated, leading to *de novo *synthesis of several cytokines and chemokines [24]. Results presented herein showed that in diabetic rats the levels of IL-1β and TNF-α in the BALF were significantly lower than in non-diabetic rats under allergic stimulation. Treatment with insulin completely restored the release of IL-1β and TNF-α during the allergic lung inflammation. Indeed, insulin treatment of diabetic rats increases the levels of these cytokines [7] associated with increased expression of their transcripts in the lungs [8] during the course of lipopolysaccharide (LPS)-induced lung inflammation. Cytokines such as IL-1β and TNF-α amplify the inflammatory response by stimulating the release of chemoattractant factors by alveolar macrophages and airways epithelial cells, and the expression of adhesion molecules by the endothelium [25]. In addition to its relevance to asthma in general, TNF-α mediates recruitment of neutrophils and eosinophils during airway inflammation [26]. CINC-1, a member of IL-8 family of chemokines that stimulate neutrophil migration into the lungs, is known to be induced by IL-1β and TNF-α [27]. Despite the significantly lower concentrations of IL-1β and TNF-α in the BALF of OA challenged diabetic rats, as demonstrated in the present study, equivalent increases in the levels of CINC-1 were observed in diabetic and control rats. Furthermore, CINC-1 levels did not change after treatment of diabetic rats with insulin, which suggest that the production/release of CINC-1 might be independent of insulin. However, it is possible that 6 hours was not the time required for CINC-1 to reach the maximum release after OA challenge. Other chemokines, such as macrophage inflammatory protein (MIP)-1α, or MIP-2 (renamed CINC-3) could be responsible for the recruitment of neutrophils into the lung rather than CINC-1.

Chemokines produced locally then promote the arrest of leukocytes on the endothelium and their migration into surrounding tissues [26,27]. Thus, the migration of leukocytes into an asthmatic lung is dependent upon multiple mechanisms that are initiated by binding of leukocytes at the endothelial border to selectins [26-29]. Increased expression of E- and P-selectins has been observed in asthmatic patients [28,29], suggesting that these molecules are up-regulated in human disease and may contribute to the development of both bronchial inflammation and airway hyperreactivity. It has been shown that P-selectin deficient mice exhibit a reduction of airway hyperresponsiveness, and decreased cellular recruitment into the airways in response to OA inhalation [30]. Data, presented herein, showed that lung neutrophils infiltration occurred in parallel with the increased endothelial expression of E- and P-selectins in lung microvessels of OA-challenged rats. Unexpectedly, only a reduction in the expression of P-selectin after OA challenge was associated with reduced cellular migration that was completely restored by treatment of diabetic rats with insulin. Nevertheless, despite no significant differences between diabetic and control groups after OA-challenge, E-selectin expression on lung vessels further increased after insulin treatment of diabetic rats. These results suggest that insulin modulates E- and P-selectin in the allergic airway inflammation. Furthermore, it has been shown that insulin modulates the expression of ICAM-1 on the alveolar epithelium and the associated leukocyte infiltration during the course of allergen-induced airway inflammation in rats [31]. Indeed, insulin treatment of diabetic rats up-regulates ICAM-1 protein and gene expression in the lungs during the course of LPS-induced lung inflammation [7,8]. Regulation of ICAM-1 transcript by insulin involves, at least in part, activation of the nuclear factor (NF)-κB pathway in the lungs of diabetic rats [8], suggesting a mechanism through which insulin might modulate E- and P-selectin expression in the early phase of the allergic lung inflammation.

The ability of insulin to mediate expression of inflammatory cytokines as well as of adhesion molecules suggests a role for insulin in the regulation of inflammation. Further studies on the mechanisms of insulin action on leukocytes and endothelial cells are needed to understand its role in allergic lung inflammation.

Clinical asthma appears to be less severe when diabetes mellitus is superimposed. Data from epidemiological studies in children with type-1 diabetes mellitus [32,33] are consistent with data from previously published studies [34,35], reporting an inverse relationship between atopy and diabetes mellitus. It is possible to speculate that asthma is suppressed in diabetic individuals because there is a relative lack of insulin which, in turn, would allow asthma to manifest itself clinically. In this context, it has been already demonstrated that insulin modulates the early response to antigen provocation by controlling mast cell degranulation [5]. Data presented herein suggest that insulin modulates levels of cytokines, expression of adhesion molecules, and cell migration during the early phase of allergic airway inflammation. This effect of insulin should be primarily due to the elevated levels of insulin maintained during the experiment since blood glucose levels were only partially reduced by this protocol of insulin treatment.

## Conclusion

Data presented suggest that insulin modulates the production/release of the pro-inflammatory cytokines, TNF-α and IL-1β, the expression of P- and E-selectin, and the associated neutrophil migration into the lungs during the early phase of the allergic inflammatory reaction.

## Competing interests

The authors declare that they have no competing interests.

## Authors' contributions

JOM - designed the study, performed the measurements and animal experiment, interpreted the data and wrote the manuscript. CALC, JWMCC and VAFA - performed and analysed the immunohystochemistry. SM and EOV - performed the lung morphometric analysis. SJ and PS - designed the study, interpreted the data and helped to write the manuscript. All authors read and approved the final manuscript.

## Pre-publication history

The pre-publication history for this paper can be accessed here:

http://www.biomedcentral.com/1471-2466/10/39/prepub
